# Assessing the Utility of Over‐The‐Counter Medication to Track Influenza Season Timing in a South African Population

**DOI:** 10.1111/irv.70313

**Published:** 2026-09-08

**Authors:** Malebo Sephule Makunyane, Neville Sweijd, Jhandre Bredenkamp, Sibongile Walaza, Cheryl Cohen, Jonny Peter

**Affiliations:** ^1^ Division of Allergology and Clinical Immunology, Department of Medicine, Faculty of Health Sciences University of Cape Town Cape Town South Africa; ^2^ Allergy and Immunology Unit University of Cape Town Lung Institute Cape Town South Africa; ^3^ School for Climate Studies Stellenbosch University Stellenbosch South Africa; ^4^ Alliance for Collaboration on Climate and Earth Systems Science (ACCESS) Council for Scientific and Industrial Research Cape Town South Africa; ^5^ Clicks Retailers Clicks Group Limited Cape Town South Africa; ^6^ Centre for Respiratory Diseases and Meningitis National Institute for Communicable Diseases of the National Health Laboratory Service Johannesburg South Africa; ^7^ School of Public Health, Faculty of Health Sciences University of the Witwatersrand Johannesburg South Africa

**Keywords:** influenza, over‐the‐counter medication sales, proxy data, South Africa, surveillance

## Abstract

**Background:**

Laboratory‐based influenza surveillance in low‐ and middle‐income countries may have limited timeliness and geographic coverage. We evaluated whether over‐the‐counter (OTC) respiratory medication sales track seasonal influenza activity and provide early warning of influenza onset in South Africa.

**Methods:**

Daily OTC medication sales were obtained from a national pharmaceutical chain and analysed nationally and in Gauteng, KwaZulu‐Natal and Western Cape. Weekly laboratory‐confirmed influenza cases were obtained from sentinel surveillance programmes for 2012–2019 and 2022–2023. Seasonal characteristics were compared using the Moving Epidemic Method. Lead–lag relationships were assessed using Pearson cross‐correlation, with 3‐week smoothing and first differencing as sensitivity analyses. Forecasts at 1‐ to 5‐week horizons were evaluated using influenza‐only, OTC‐only, combined influenza‐plus‐OTC generalized linear model (GLM), Random Forest and naïve persistence models. Performance was assessed using root mean square error and area under the receiver operating characteristic curve (AUC).

**Results:**

OTC sales preceded increases in influenza detections nationally and provincially. Sales‐defined seasons began and peaked earlier in 70% of national seasons and 67%–89% across provinces. Primary analyses indicated that OTC sales preceded influenza detections by 2–3 weeks (mean peak correlations, 0.77–0.81). After first differencing, OTC changes preceded influenza changes in 5–8 of 10 seasons across locations (correlations, 0.42–0.50). Influenza‐only models produced the lowest forecast errors. OTC‐informed models achieved AUC ≥ 0.90 at 1‐ to 2‐week horizons. All significant squared‐error comparisons favoured naïve persistence.

**Conclusions:**

OTC respiratory medication sales may complement virological surveillance by supporting earlier identification of seasonal influenza activity but should not replace laboratory surveillance.

## 1 Introduction

Respiratory viral infections, including influenza, pose a substantial burden to public health systems because of their high transmissibility and potential to cause severe complications in vulnerable populations [1, 2]. The World Health Organization defines influenza‐like illness (ILI) as an acute respiratory infection characterized by a fever of ≥ 38°C and cough, with symptom onset within the past 10 days [3]. ILI may be caused by a range of respiratory viruses, including but not limited to influenza viruses. Influenza itself is a distinct viral infection that may present as uncomplicated disease or progress to severe illness requiring hospitalization, particularly when lower respiratory tract involvement is present [4].

It is estimated that between 6734 and 11,619 seasonal influenza‐associated deaths occur annually in South Africa, with the highest burden among children under five, the elderly and individuals living with HIV [5]. Among patients presenting with ILI, approximately 25% test positive for influenza by polymerase chain reaction (PCR), underscoring the limited specificity of syndromic case definitions and the diagnostic challenges posed by overlapping clinical presentations of respiratory infections [6, 7].

In South Africa, influenza activity typically coincides with the winter period, most frequently between epidemiological weeks 16 and 25 (April to June), with peak activity observed from mid‐May (around Week 20) to early July. However, substantial interannual variability in season onset and peak timing has been documented, with a mean season onset around Week 17 and a reported range from 16 to 25 [8, 9]. In 2025, influenza activity began earlier than usual, with onset in Week 13 (24 March), followed by a peak in Week 20 (12 May) and an end of the season in Week 30 (21 July) [10]. This unusually early onset emphasizes the importance of systems capable of rapidly detecting deviations from expected seasonal timing to support health system preparedness.

Globally, a variety of tools have been utilized for syndromic surveillance for early detection of outbreaks [11, 12], including OTC medication sales [13, 14], internet search queries (e.g., Google Trends) [15] and electronic records for ambulances [16]. These systems aim to capture early signals of community transmission, enabling timely public health action. Although South Africa has a well‐established laboratory‐based influenza surveillance system, testing is conducted primarily through sentinel platforms and is not routinely performed for all patients presenting with respiratory illness. Consequently, complementary data sources, such as pharmaceutical medication sales, may provide additional information on community respiratory illness activity and improve the timeliness of surveillance.

OTC medications commonly used to manage symptoms of ILI, such as antipyretics, decongestants, cough suppressants and antihistamines, may reflect the burden of mild, community‐level respiratory illness prior to individuals seeking clinical care [17]. Moreover, these data are routinely collected, geocoded and updated in near‐real time, enhancing their utility for high‐resolution temporal monitoring.

Despite growing interest in pharmaceutical sales data for syndromic surveillance, few studies in Africa have evaluated their temporal relationship with laboratory‐confirmed influenza activity. To address this gap, this study examined whether national and provincial OTC medication sales followed seasonal patterns consistent with laboratory‐confirmed influenza detections, whether they provided earlier signals of influenza activity and how closely their temporal dynamics aligned with established surveillance indicators. Because OTC respiratory medications are used for a range of respiratory illnesses, the objective was not to evaluate influenza‐specific medication use but rather to assess whether aggregated sales could serve as a complementary proxy for influenza surveillance. We hypothesized that individuals experiencing early respiratory symptoms may initially self‐medicate before seeking clinical care, resulting in increases in OTC medication sales that precede laboratory‐confirmed influenza detections. Accordingly, the primary aim of this study was to assess whether provincial OTC medication sales could serve as an early complementary indicator of laboratory‐confirmed influenza activity in South Africa and support syndromic surveillance.

## 2 Methodology

### 2.1 Study Design and Methodology

This study employed a retrospective ecological time‐series design covering the period from 8 January 2012 to 30 September 2023. Data from 2020 and 2021 were excluded because non‐pharmaceutical interventions and disruptions to healthcare utilization during the COVID‐19 pandemic substantially altered both influenza transmission and surveillance patterns, rendering these years non‐comparable to typical seasonal influenza dynamics [18, 19].

**FIGURE 1:**
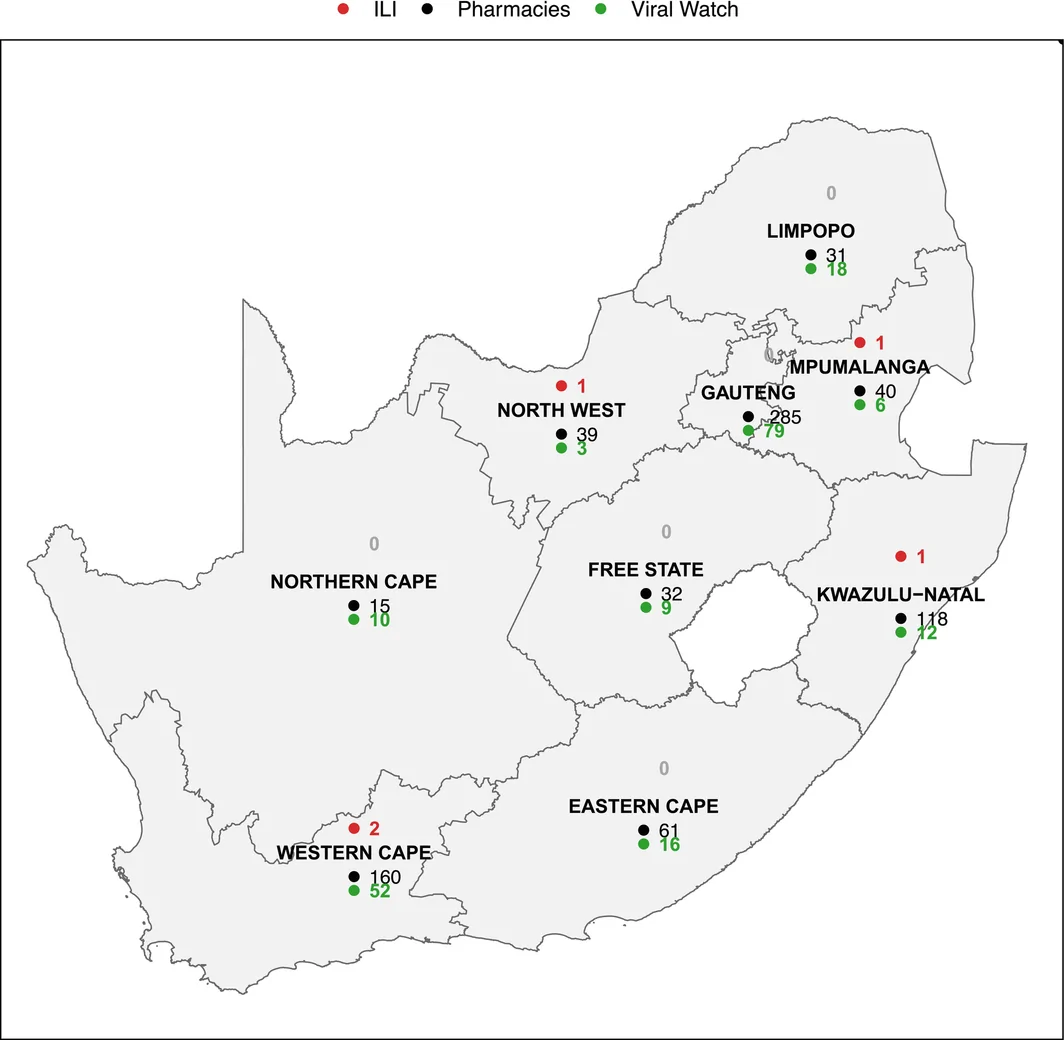
Map showing numbers of Viral Watch and ILI sentinel sites and pharmacy stores across South Africa.

The analysis investigated associations between over‐the‐counter (OTC) medication sales and laboratory‐confirmed influenza activity at national level (aggregate of all nine provinces) and across three South African provinces, namely, Gauteng, KwaZulu‐Natal and Western Cape (Figure 1). These provinces were selected because they had the highest and most consistent volumes of laboratory‐confirmed influenza cases within the sentinel surveillance system during the study period, enabling stable seasonal estimation and robust lead–lag analysis. OTC sales data were available across all provinces; however, analysis was restricted to provinces with sufficient laboratory‐confirmed influenza activity to support reliable comparative modelling.

### 2.2 OTC Medication Sales Data

Daily OTC medication sales data were obtained from a large national retail pharmacy chain in South Africa. The number of in‐store pharmacies increased from 123 in 2012 to 781 by the beginning of 2025, resulting in progressively broader geographic coverage over time. Each record contained aggregated daily counts of respiratory‐related OTC medications sold per province. OTC respiratory medication sales were derived from a predefined basket of 319 respiratory‐related OTC products identified by the pharmacy retailer. These products were grouped into therapeutic categories, including cold and influenza combination remedies, cough suppressants, expectorants and mucolytics, chest congestion remedies, nasal decongestants, sore throat preparations, paediatric respiratory formulations and bronchial symptom treatments. Representative active ingredients and product counts by therapeutic category are provided in Table S1. Daily sales counts were aggregated to weekly totals for each province for analysis. National‐level sales were calculated as the sum of weekly sales across all nine provinces.

### 2.3 Influenza Surveillance Data

Influenza data were obtained from the National Institute for Communicable Diseases (NICD) and derived from two long‐standing sentinel surveillance programmes, namely, the ILI programme implemented in public primary healthcare clinics and the Viral Watch (VW) programme, which monitors influenza activity through private general practitioner (GP) practices [6]. The VW programme, established in 1984, is a voluntary, clinic‐based surveillance system designed to track outpatient influenza trends. Figure 1 shows the coverage map of the surveillance programmes and pharmacies across South Africa. Combined, the two surveillance programmes operate across 146 sentinel sites in the three provinces [6], specifically 79 VW in Gauteng, 12 VW and one ILI in KwaZulu‐Natal and 52 VW and two ILI in Western Cape. Influenza activity was defined as the weekly count of laboratory‐confirmed influenza virus detections aggregated across sentinel sites within each province. For analysis, we combined the ILI and VW influenza detection data within each province.

### 2.4 Data Preprocessing

To reduce week‐to‐week variability while preserving underlying seasonal trends, similar to previous studies [20, 21], we applied a 7‐week centred moving average (MA7) to both influenza detections and OTC sales time series for primary analyses. This smoothing window was selected to balance noise reduction in routine surveillance data with retention of epidemiological variation in peak timing and magnitude. Sensitivity analyses were conducted using 3‐week (MA3) and 5‐week (MA5) moving averages to assess the robustness of results to different levels of temporal smoothing, consistent with approaches used by NICD and CDC influenza surveillance programmes [19, 22]. Smoothed series were only used for seasonal analyses and cross‐correlation. Forecasting analyses were performed using the original weekly aggregated data after standardization and did not use moving‐average smoothing.

Smoothed series were used only for the MEM and cross‐correlation analyses. Forecasting analyses used the original unsmoothed weekly series. Continuous predictor variables were standardized using the mean and standard deviation estimated from the training data. The same parameters were applied to the testing data. The forecast outcome, weekly influenza detections, remained on the original count scale.

### 2.5 Influenza Season Definition Using the Moving Epidemic Method (MEM)

To compare the timing and characteristics of influenza and OTC medication sales seasons, including onset, duration, peak timing and intensity, the MEM was applied [23]. MEM calculates epidemic and intensity thresholds from historical data using confidence intervals based on upper limits of pre‐epidemic thresholds [23]. Epidemic periods were defined as intervals during which the smoothed influenza activity exceeded the epidemic threshold for at least two consecutive weeks [23, 24].

### 2.6 Cross‐Correlation Analysis

To examine the temporal relationship between OTC medications and influenza activity, Pearson cross‐correlation analyses were performed using the standardized MA7‐smoothed series [25, 26]. Cross‐correlations were estimated over lag periods ranging from −8 to +8 weeks. Negative lags indicated that changes in OTC medication sales preceded corresponding changes in influenza detections. The analysis focused on identifying lead or lag structures relevant to early warning potential, where negative lags indicate OTC sales leading subsequent increases in influenza detections.

For sensitivity analysis, first‐differenced time series were used to assess the potential influence of shared seasonal trends on observed correlations [27, 28]. Influenza detections and OTC sales were smoothed using a 3‐week centred moving average, after which week‐to‐week changes were calculated by differencing consecutive observations and standardizing the resulting series. Cross‐correlation analyses were then repeated using the differenced series. Consistency between the primary and sensitivity analyses would support the interpretation that observed lead–lag relationships were not solely attributable to shared seasonal trends.

### 2.7 Short‐Term Forecasting Framework

Short‐term forecasting models adapted from prior comparative modelling studies [25, 29] were fitted to evaluate whether OTC medication sales improved the prediction of influenza activity at 1‐ to 5‐week horizons. For each horizon, the outcome was defined as influenza activity h weeks ahead. Predictors included recent influenza activity and lagged OTC sales. Five models were evaluated: (i) a combined influenza‐plus‐OTC generalized linear model (GLM) with current and autoregressive influenza terms, (ii) a flu‐only GLM and (iii) OTC‐only GLM following the approach of Ohkusa et al. [25], (iv) a Random Forest regression model and (v) a naïve persistence model assuming that future influenza activity was equal to the most recent observation.

Let $Y_{t}$ denote influenza detections in week t, $X_{t}$ denote OTC sales and h∈1,2,3,4,5 denote the forecast horizon.

Combined GLM:

(1) $$g\left(\mu_{t+h}\right)=\beta_{0}+\sum_{j=0}^{2}\alpha_{j}Y_{t-j}+\sum_{j=0}^{2}\gamma_{j}X_{t-j}$$

Flu‐only GLM:

(2) $$g\left(\mu_{t+h}\right)=\beta_{0}+\sum_{j=0}^{2}\alpha_{j}Y_{t-j}$$

OTC‐only GLM:

(3) $$g\left(\mu_{t+h}\right)=\beta_{0}+\sum_{j=0}^{2}\gamma_{j}X_{t-j}$$

Random Forest

$$z_{t}=\left(Y_{t},Y_{t-1},Y_{t-2},X_{t},X_{t-1},X_{t-2}\right)$$

Then:

(4) $${\hat{Y}}_{t+h}=\frac{1}{B}\sum_{b=1}^{B}T_{b}\left(z_{t}\right)$$

Naïve persistence model

(5) $${\hat{Y}}_{t+h}=Y_{t}$$

For each province and forecast horizon, observations were ordered chronologically and split into training (first 80%) and testing (remaining 20%) sets. Predictive accuracy was assessed using the root mean squared error (RMSE) and mean absolute error (MAE). The incremental value of OTC sales was assessed by comparing the combined models with flu‐only GLM and naïve persistence models.

Early‐warning performance was evaluated by classifying high‐influenza weeks, defined as weeks exceeding the 75th percentile of the training distribution, and quantified using area under the receiver operating characteristic curve (AUC). The AUC was defined as

(6) $$\mathrm{AUC}=\mathrm{Pr}\left(S_{H}>S_{L}\right)+\frac{1}{2}\mathrm{Pr}\left(S_{H}=S_{L}\right)$$

Here, $S_{H}$ and $S_{L}$ denote predicted influenza‐activity scores for randomly selected high‐ and low‐influenza weeks, respectively. An AUC of 0.5 indicates no discrimination beyond chance, whereas an AUC of 1.0 indicates perfect discrimination. At each forecast horizon and geographical level, each model was compared with the naïve persistence model. Differences in AUC were assessed using paired DeLong tests. Differences in forecast accuracy were assessed using Diebold–Mariano tests with squared‐error loss. All analyses were conducted in R (version 4.5.2).

## 3 Results

### 3.1 Seasonal Patterns in Influenza Detections and OTC Respiratory‐Medication Sales

**FIGURE 2:**
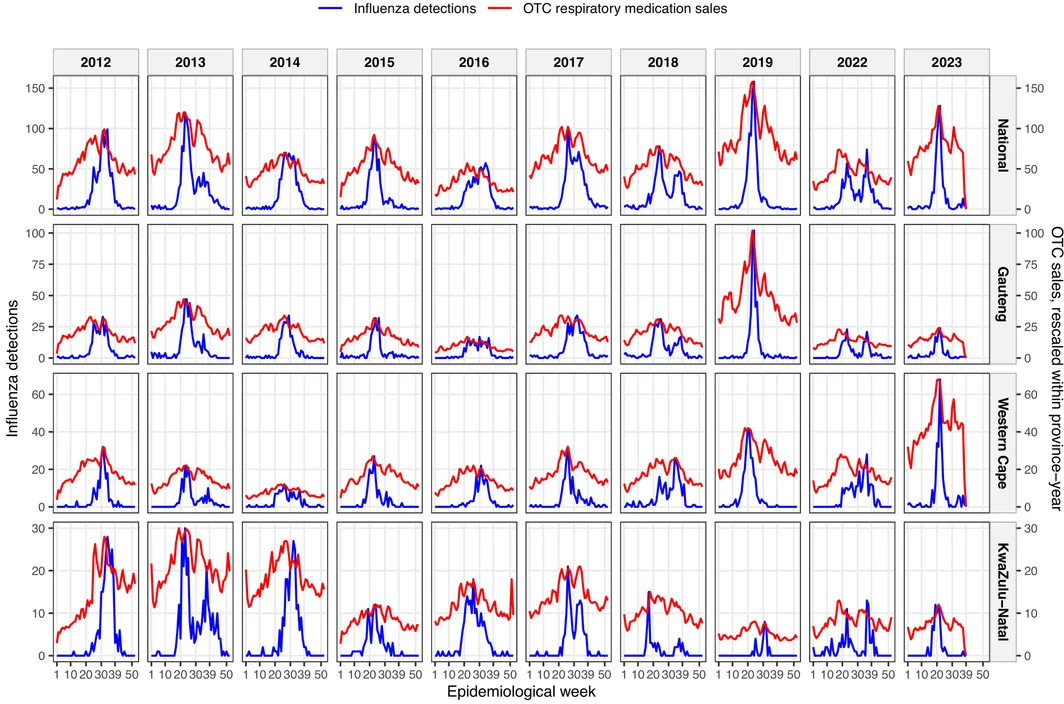
Weekly influenza detections (blue) and OTC respiratory‐medication sales (red) by epidemiological week, 2012–2019 and 2022–2023 (excluding 2020–2021). Panels show series for national, Gauteng (GP), Western Cape (WC) and KwaZulu‐Natal. OTC sales were rescaled within each province‐year for visualization only. Rescaled values represent relative within‐year sales intensity and were not used in the statistical analyses.

Figure 2 displays weekly laboratory‐confirmed influenza detections and rescaled OTC respiratory‐medication sales for the national series, Gauteng (GP), Western Cape (WC) and KwaZulu‐Natal (KZN) from January 2012 to September 2023, excluding 2020 and 2021. Across all settings, both indicators show clear annual seasonal rises and declines, with single dominant peaks in most years and more complex multi‐peak patterns in 2016, 2017, 2018 and 2023. The national series exhibits consistently narrow influenza peaks and broader sales curves across all years. Gauteng shows variable annual patterns, with several seasons (2013, 2016, 2017, 2018) characterized by two distinct increases in both indicators. Western Cape displays relatively sharp influenza peaks and smoother, wider sales curves, with noticeable year‐to‐year variation in amplitude. KwaZulu‐Natal shows greater variability in influenza detections, including periods with secondary rises, while sales curves remain smoother and more continuous across years. Week‐of‐year climatology similarly shows that both laboratory‐confirmed influenza detections and OTC respiratory medication sales exhibit consistent winter seasonal patterns across the study period (Figure S1).

### 3.2 MEMs

**FIGURE 3:**
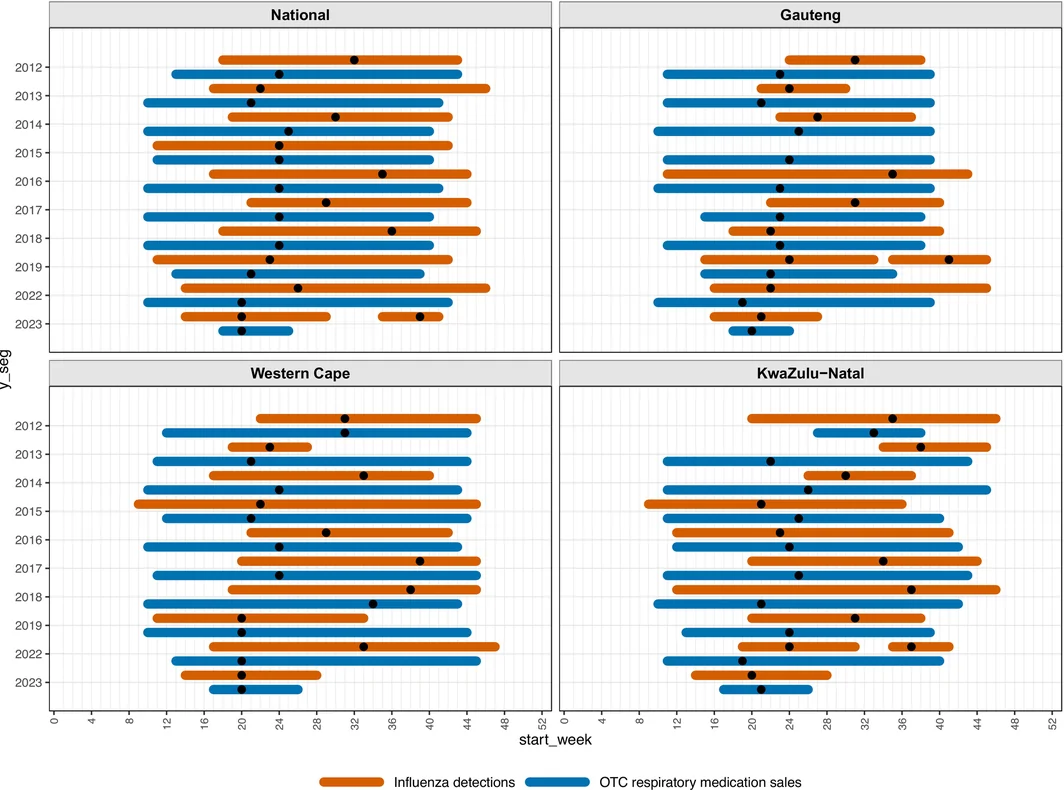
Season windows (bars) and peak weeks (dots) for influenza detections (orange) and over‐the‐counter (OTC) respiratory medication sales (blue), by epidemiological week, 2012–2019 and 2022–2023. Panels show National, Gauteng, KwaZulu‐Natal and Western Cape. Bars span the start–end of each MEM‐defined season based on 7‐week moving averages (MA7); dots mark the peak week within each season. Years are displayed as rows (earliest at top), with separate lanes for influenza (upper) and sales (lower).

Figure 3 shows seasonal windows (bars) and peak weeks (dots) for influenza detections (red) and OTC respiratory‐medication sales (blue) in National, Gauteng, KwaZulu‐Natal and Western Cape, 2012–2019 and 2022–2023. Nationally, comparing the start, duration, peak and intensity of the influenza season using both OTC sales and influenza detection rate. Sales‐based seasons started earlier in 7/9 years (coincided in 1/9 and started later in 1/9). Sales peaks occurred earlier in 8/9 years (coincided in 1/9 of years). Sales seasons ended earlier in 8/9 years (coincided in 1/9 years). Sales windows were longer than influenza‐defined seasons in 6/9 years, and in multi‐wave influenza seasons, sales retained a single broad seasonal window.

In Gauteng, sales‐defined seasons started earlier in 8/9 years (coincided in 1/9 years). Peaks in sales occurred earlier in 8/9 years (started later in 1/9 years). Seasonal end dates occurred earlier in 5/9 years (occurred later in 4/9 years). Sales seasons were longer in 7/9 years. In KwaZulu‐Natal, sales seasons began earlier in 7/10 years (coincided in 1/10 and started later in 1/10). Sales peaks occurred earlier in 7/10 years (started later in 2/10 years). Sales seasons ended earlier in 4/10 years and ended later in 5/10. Sales‐based seasons were longer in 8/10 years. In the Western Cape, sales seasons started earlier in 8/10 years and coincided in 2/10. Peaks in sales occurred earlier in 7/10 years and coincided in 3/10. Seasonal end dates occurred earlier in 5/10 years, coincided in 1/10 and ended later in 4/10. Sales seasons were longer in 8/10 years.

### 3.3 Cross‐Correlation and Time‐Lead Between Flu Activity and Medication Sales

**FIGURE 4:**
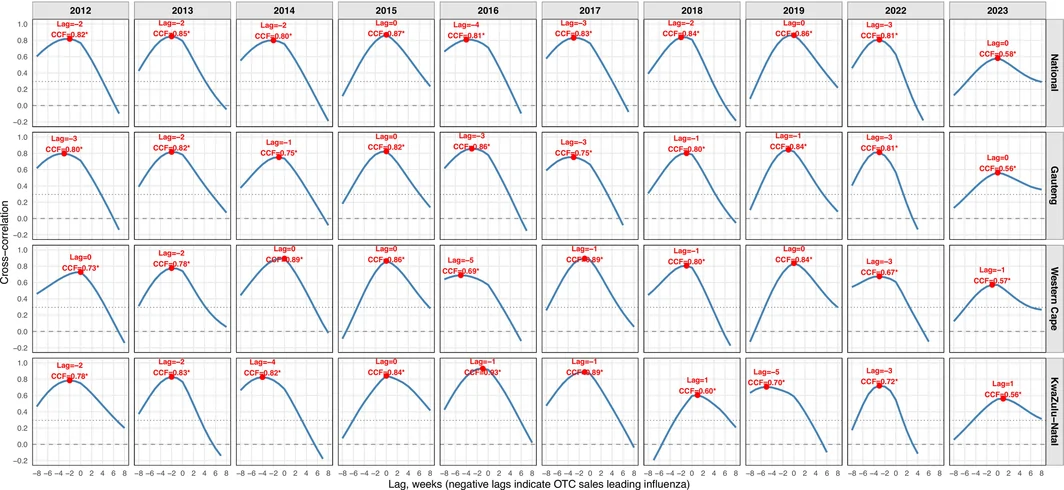
Cross‐correlation between OTC respiratory medication sales and influenza detections by province and year (2012–2019, 2022–2023). Year‐specific cross‐correlation functions (CCFs) are shown for the national dataset and three provinces. Negative lags indicate that OTC sales preceded influenza detections. Red points denote annual peak CCF values with corresponding lag labels. Weekly series were smoothed using a 7‐week moving average.

Across the study period (2012–2019 and 2022–2023), peaks in OTC respiratory medication sales generally preceded peaks in influenza detections, with strong annual cross‐correlation coefficients in most years across all provinces (Figure 4). Table 1 summarizes the number of years in which OTC sales led influenza activity, the range and mean of leading lags and the mean peak cross‐correlation across seasons with corresponding 95% confidence intervals.

**TABLE 1:** Summary of lead–lag relationships between OTC respiratory medication sales and influenza detections by province, including mean peak cross‐correlation coefficients (95% confidence intervals) across seasons.

Province	Number lead	Lead range	Mean lead	Mean peak
National	7/10	−4 to −2	−2.57	0.806 (0.747; 0.865)
Gauteng	8/10	−3 to −1	−2.12	0.781 (0.721; 0841)
Western Cape	6/10	−5 to −1	−2.17	0.773 (0.697; 0.850)
KwaZulu‐Natal	7/10	−5 to −1	−2.57	0.768 (0.682; 0.853)

At the national level, OTC sales led influenza activity in 7 of 10 years, with leading lags ranging from −4 to −2 weeks and a mean lead of −2.57 weeks. The mean peak cross‐correlation coefficient across seasons was 0.806 (95% CI: 0.747–0.865). The strongest annual correlation was observed in 2015 (CCF = 0.868). In Gauteng, OTC sales led influenza activity in 8 of 10 years, with peak lags between −3 and −1 weeks and a mean lead of −2.12 weeks. The mean peak cross‐correlation coefficient was 0.781 (95% CI: 0721–0841), with the strongest annual correlation observed in 2016 (CCF = 0.856, at a lag of −2 weeks). In the Western Cape, OTC sales led influenza activity in 6 of 10 years, with leading lags ranging from −5 to −1 weeks and a mean lead of −2.17 weeks. The mean peak cross‐correlation coefficient was 0.773 (95% CI: 0.697–0.850). The strongest annual correlation occurred in 2017 (CCF = 0.895, at a lag of −3 weeks). In KwaZulu‐Natal, OTC sales led influenza activity in 7 of 10 years, with peak lags between −5 and −1 weeks and a mean lead of −2.57 weeks. The mean peak cross‐correlation coefficient was 0.768 (95% CI: 0.682–0.853), while the strongest annual correlation was observed in 2016 (CCF = 0.930, at a lag of −3 weeks).

Sensitivity analyses using first‐differenced series yielded results broadly consistent with the primary analyses (Table S2 and Figure S2). OTC sales changes preceded influenza changes in 7/10 seasons nationally, 8/10 seasons in Gauteng, 6/10 seasons in Western Cape and 5/10 seasons in KwaZulu‐Natal. Mean peak cross‐correlations ranged from 0.424 (95% CI: 0.355–0.492) in KwaZulu‐Natal to 0.503 (95% CI: 0.421–0.584) in Western Cape, indicating that the observed lead–lag relationships remained evident after reducing the influence of shared seasonal trends.

### 3.4 Short‐Term Forecasting

**FIGURE 5:**
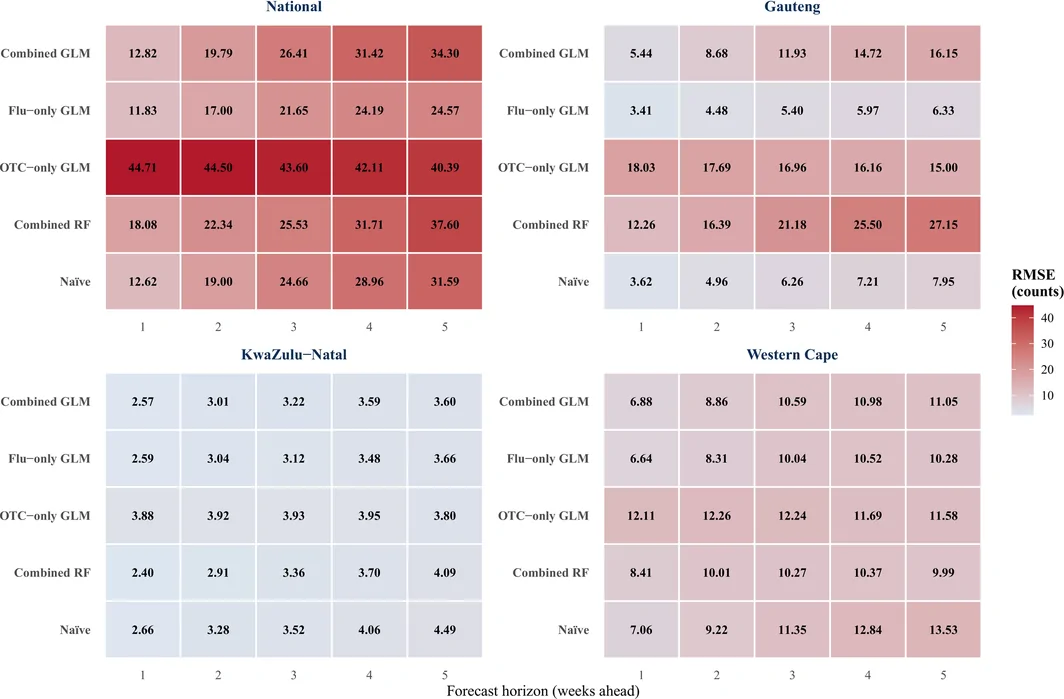
Forecast error (RMSE) for influenza predictions using OTC medication sales across provinces and forecast horizons.

Figure 5 presents the RMSE of weekly influenza detection at forecast horizons of one to 5 weeks at national and provincial levels. Across all models and geographic scales, forecast error increased with longer horizons. The flu‐only GLM consistently achieved the lowest or near‐lowest RMSE across most settings. At national level, mean RMSE for the flu‐only model ranged from 11.93 at 1 week ahead to 24.78 at 5 weeks ahead. Similar patterns were observed in Gauteng (3.43–6.43), Western Cape (6.70–10.37) and KwaZulu‐Natal (2.62–3.71). At the 1‐week horizon, the naïve persistence model achieved the lowest RMSE in all settings, with values of 0.47 nationally, 0.31 in Gauteng, 1.02 in Western Cape and 0.45 in KwaZulu‐Natal. However, its performance deteriorated rapidly, with RMSE exceeding 1.0 by week four in the national and Western Cape series.

Models incorporating both influenza activity and OTC medication sales did not consistently improve the forecast accuracy. Compared with the flu‐only GLM, the combined GLM produced higher RMSE across most forecast horizons nationally, in Gauteng and in the Western Cape, while performance in KwaZulu‐Natal was comparable. The combined Random Forest model generally produced the highest forecast error, particularly at longer forecast horizons. OTC‐only models consistently yielded larger prediction errors than models incorporating influenza activity.

**FIGURE 6:**
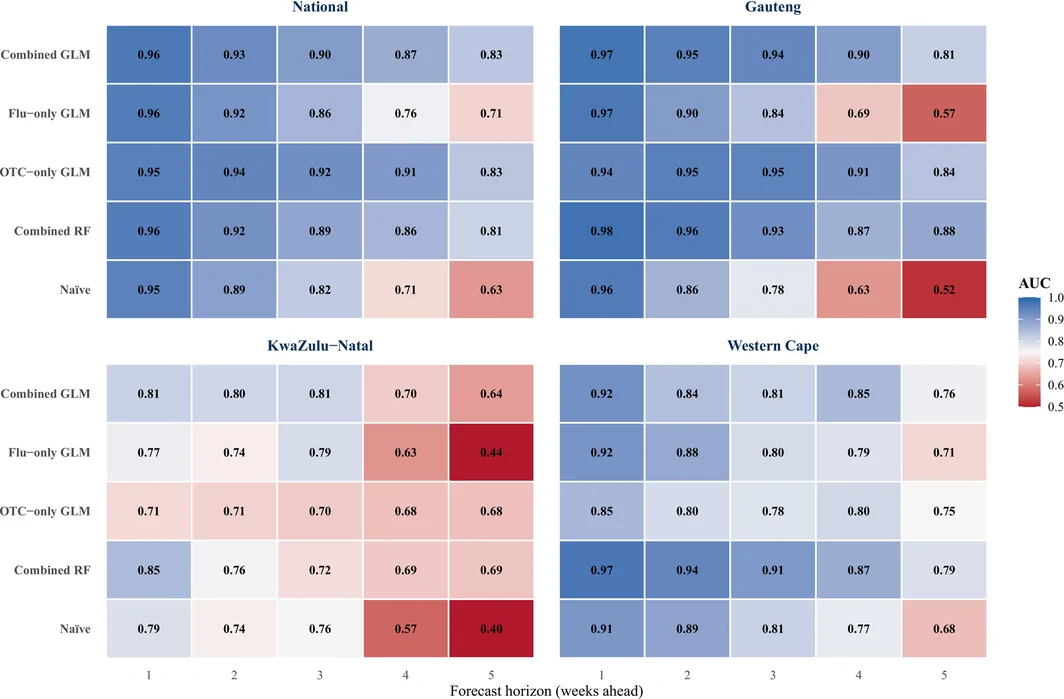
Early detection performance (AUC) for identifying high‐influenza weeks up to 5 weeks in advance.

Figure 6 summarizes model discrimination of high influenza activity weeks using the AUC. Nationally, the combined GLM, combined Random Forest and OTC‐only GLM maintained acceptable and good discrimination through 5 weeks. The flu‐only GLM maintained acceptable discrimination through 5 weeks and good discrimination through 3 weeks, whereas the naïve model maintained these thresholds through four and 3 weeks, respectively. Similar sustained discrimination was observed for the OTC‐informed models in Gauteng and the Western Cape, whereas performance was lower and more variable in KwaZulu‐Natal. Pairwise DeLong comparisons with the naïve persistence model showed that several OTC‐informed models achieved significantly higher AUCs at selected forecast horizons (Figure S3). In contrast, Diebold–Mariano tests showed no significant reduction in squared‐error loss for OTC‐informed models; all significant comparisons favoured the naïve model after Benjamini–Hochberg adjustment (Figure S4).

Table 2 presents the maximum forecast horizons achieving acceptable (AUC ≥ 0.70) and good (AUC ≥ 0.80) discrimination. In Gauteng, the combined GLM, combined Random Forest and OTC‐only GLM achieved both thresholds through 5 weeks. In KwaZulu‐Natal, the combined GLM achieved both thresholds through 3 weeks, whereas the combined Random Forest achieved acceptable discrimination through 3 weeks and good discrimination through 1 week. In the Western Cape, the combined GLM, combined Random Forest and OTC‐only GLM achieved acceptable discrimination through 5 weeks and good discrimination through 4 weeks.

**TABLE 2:** Maximum forecast horizon at which models achieved acceptable and good discrimination.

Province	Model	AUC ≥ 0.70	AUC ≥ 0.80
National	Combined GLM	5	5
	Combined Random Forest	5	5
	Flu‐only GLM	5	3
	Naïve persistence	4	3
	OTC‐only GLM	5	5
Gauteng	Combined GLM	5	5
	Combined Random Forest	5	5
	Flu‐only GLM	3	3
	Naïve persistence	3	2
	OTC‐only GLM	5	5
KwaZulu‐Natal	Combined GLM	3	3
	Combined Random Forest	3	1
	Flu‐only GLM	3	—
	Naïve persistence	3	—
	OTC‐only GLM	2	—
Western Cape	Combined GLM	5	4
	Combined Random Forest	5	4
	Flu‐only GLM	5	2
	Naïve persistence	4	3
	OTC‐only GLM	5	4

## 4 Discussion

This study evaluated whether OTC medication sales for cough and cold capture temporal patterns consistent with laboratory‐confirmed influenza detections in South Africa, and whether they provide early signals of seasonal influenza dynamics. Using more than a decade of data and multiple complementary analytic methods, including seasonal window analysis, cross‐correlation and forecasting models, OTC sales showed a close temporal association with influenza detections and increases in OTC sales typically preceded increases in laboratory‐confirmed influenza by approximately 2–3 weeks. These findings indicate that routinely collected pharmaceutical sales data can function as a useful supplementary indicator of community respiratory illness activity and may support influenza surveillance, particularly in settings where laboratory surveillance has limited timeliness or coverage.

### 4.1 OTC and Influenza Seasonal Patterns

Seasonal analysis based on the MEM showed that OTC respiratory‐medication sales consistently defined seasons that began earlier, peaked earlier and lasted longer than influenza seasons defined by laboratory detections. Several mechanisms likely contribute to these differences. First, increases in OTC sales are expected to occur soon after symptom onset, as individuals with mild illness frequently self‐medicate before seeking clinical care or undergoing diagnostic testing. Second, OTC sales capture a broader spectrum of respiratory symptom burden, including non‐influenza viral infections and lingering post‐infectious symptoms, which may extend beyond the period of virologically confirmed influenza circulation. Third, aggregation of multiple overlapping respiratory drivers likely produces smoother seasonal curves and a single broad peak in sales, even in years when influenza activity had more than one wave.

In several seasons with weak or multi‐peak influenza epidemics, particularly in Gauteng and KwaZulu‐Natal, laboratory detections appeared discontinuous, whereas OTC sales showed a more continuous seasonal rise. This suggests that sales data may reflect underlying community respiratory activity or be confounded by aeroallergen‐driven symptom exacerbations, although this requires further investigation. Similar findings have been reported in pharmacy‐based seasonal analyses in Europe and Asia, where community pharmacy data have provided earlier and more stable seasonal signals than traditional surveillance indicators alone [25, 30].

### 4.2 Temporal Alignment and Correlation

Cross‐correlation analyses demonstrated that OTC sales generally led influenza detections by approximately 2–3 weeks, with strong peak correlation coefficients. These results are consistent with studies conducted in Japan, the United States and Europe, where pharmaceutical purchases have been shown to precede outpatient ILI consultations and laboratory‐confirmed influenza cases [25, 31, 32, 33, 34]. For example, Magruder et al. reported that OTC medications commonly used for influenza led outpatient respiratory diagnoses by 1–3 weeks [34]. Ohkusa et al. in Japan found that sales patterns were highly correlated with influenza activity and provided a real‐time signal suitable for epidemic monitoring, with increases in OTC sales preceding rises in clinical influenza reports [25]. These observations support the use of OTC medication sales as a proxy for broader community respiratory illness.

Because both influenza activity and OTC respiratory medication sales exhibit strong winter seasonality [27, 28], part of the observed association is expected to reflect common seasonal forcing. However, sensitivity analyses based on first‐differenced time series demonstrated that OTC sales changes generally continued to precede influenza changes in most seasons (Table S2 and Figure S2) [27]. These findings suggest that the observed lead–lag relationships are not solely attributable to shared seasonal trends. Nevertheless, OTC respiratory medication sales should be interpreted as a proxy for broader respiratory illness activity rather than an influenza‐specific indicator [32], as purchasing behaviour is likely influenced by multiple respiratory pathogens and symptom complexes.

Lower and more variable correlations observed in KwaZulu‐Natal after 2018 may reflect greater interannual variability in influenza activity, including weaker or irregular peaks that reduce temporal association between sales and laboratory detections [35]. Differences in pharmacy access, healthcare‐seeking behaviour and the completeness of surveillance reporting may also contribute to regional heterogeneity in the observed correlations [36]. Across provinces, correlations were generally lower in the post‐pandemic period, likely reflecting disruptions to respiratory virus circulation, altered healthcare utilization and changes in self‐medication behaviour following the COVID‐19 pandemic [37, 38].

### 4.3 Short‐Term Forecasting

Short‐term forecasting analyses showed that performance differed according to the forecasting objective. OTC‐informed models achieved acceptable‐to‐good discrimination of future high‐influenza weeks across several locations and forecast horizons. Pairwise DeLong tests showed that several OTC‐informed models achieved significantly higher AUCs than the naïve persistence model at selected horizons after Benjamini–Hochberg adjustment. However, OTC‐informed models did not consistently improve the prediction of absolute weekly influenza detections. Diebold–Mariano tests showed no significant reduction in squared‐error loss for OTC‐informed models, and all significant comparisons favoured the naïve persistence model. RMSE comparisons also indicated that the flu‐only GLM frequently produced the lowest prediction error, particularly nationally and in Gauteng [30, 39].

These findings align with international studies, such as those using Italian retail baskets and Portuguese pharmacy data [30, 39], demonstrating that retail data have the potential to enhance the timeliness of epidemic monitoring when combined with established surveillance systems. In the South African setting, laboratory delays and uneven coverage limit early awareness; OTC sales offer a feasible complementary system that uses routinely collected data, available in near real time and geographically spread as compared to traditional surveillance systems [6, 30, 39].

### 4.4 Limitations and Strengths

This study has a few important limitations. First, OTC medication sales do not distinguish between influenza and other respiratory conditions, including rhinovirus, respiratory syncytial virus, adenovirus and aeroallergen‐driven symptoms. As a broad basket of cough and cold products was used, the sales signal likely reflects the wider winter respiratory virus season rather than influenza‐specific transmission alone. Second, sales patterns may also be influenced by factors unrelated to illness, such as seasonal purchasing behaviour, marketing stock availability, or changes in consumer preferences. Third, although sensitivity analyses indicated that the lead–lag relationship persisted after accounting for shared seasonal trends, residual confounding by co‐circulating respiratory pathogens cannot be excluded. Fourth, the use of sales data from a single retail pharmacy chain may limit representativeness despite broad geographic coverage, and expansion of store number over time may introduce temporal bias. Fifth, although smoothing was used primarily for seasonal analyses, it may have modestly strengthened the apparent temporal association between OTC sales and influenza detections. In addition, sentinel surveillance data do not represent population‐level incidence, and forecasting performance was evaluated retrospectively rather than in real‐time operation. Furthermore, exclusion of COVID‐19 pandemic years further limits temporal continuity across the study period.

Despite these limitations, this study has several strengths. It analyses more than a decade of routinely collected influenza surveillance data alongside OTC respiratory‐medication sales across multiple locations, capturing seasons with diverse timing and intensity. Secondly, the application of complementary analytic frameworks, including seasonal threshold estimation, temporal correlation analysis and short‐term forecasting, provides complementary evidence supporting the observed associations. Thirdly, the fine temporal resolution and independence of OTC sales data from clinical testing practices enabled assessment of community respiratory illness dynamics that are not fully captured by laboratory surveillance alone. Lastly, to our knowledge, this is the first study in Africa to systematically evaluate OTC respiratory‐medication sales as an early‐warning indicator for influenza using these combined methods.

### 4.5 Future Directions

Future work should investigate the drivers of secondary elevations in OTC sales, including the role of co‐circulating respiratory viruses, aeroallergen exposure and behavioural patterns of self‐medication. Integration of OTC data with a wider breadth of pathogen‐specific diagnostics, e.g., private laboratory data, environmental monitoring and additional high‐resolution data sources such as digital search trends, absenteeism records and telemedicine utilization, may further enhance early detection and forecasting performance. Prospective implementation studies are needed to evaluate the timeliness, specificity of real‐time data pipelines, privacy considerations, routine access to real‐time OTC sales data, alert thresholds and operational integration of OTC‐based signals into national surveillance systems.

## 5 Conclusion

OTC respiratory‐medication sales capture temporal changes in community respiratory illness and show strong temporal alignment with laboratory‐confirmed influenza activity in South Africa. OTC‐informed models showed potential for identifying future high‐influenza weeks but did not consistently improve prediction of absolute weekly influenza detections. OTC sales should therefore be considered a supplementary early‐warning indicator rather than a replacement for virological surveillance.

## Author Contributions

**Malebo Sephule Makunyane:** conceptualization, methodology, data curation, formal analysis, visualization, writing – original draft, writing – review and editing, validation, software, investigation, project administration. **Neville Sweijd:** conceptualization, funding acquisition, supervision, writing – review and editing, project administration. **Jhandre Bredenkamp:** data curation, resources, writing – review and editing. **Sibongile Walaza:** data curation, writing – review and editing. **Cheryl Cohen:** conceptualization, methodology, data curation, writing – review and editing. **Jonny Peter:** conceptualization, methodology, supervision, funding acquisition, resources, writing – review and editing.

## Funding

M.S.M. received financial support from the Wellcome Trust (Grant No. 311673) and the University of Cape Town Lung Institute Postdoctoral Research Fellow Award. M.S.M. and N.S. also received financial support from the National Research Foundation (NRF) Global Change Grand Challenge: Earth System Science Research Programme (ESSRP), Application of Knowledge for Management of Extreme Climate Events (APECX) (Grant No. 136477). Sentinel surveillance received funding from the National Institute for Communicable Diseases (NICD) of the National Health Laboratory Service (NHLS) and was supported in part by the US Centers for Disease Control and Prevention (CDC), Atlanta, Georgia, USA (Cooperative Agreement Nos. U51/IP000155 and NU51IP000930) and the Bill & Melinda Gates Foundation (Grant No. INV‐018978). J.P. is supported by a National Institute for Health Research (NIHR, United Kingdom) Global Research Professorship.

## Disclosure

No material requiring permission to reproduce from other sources has been included in this manuscript.

## Ethics Statement

Ethical approval was granted by the University of Cape Town Faculty of Health Sciences Human Research Ethics Committee (HREC ref. 646/2025). The study used anonymised, aggregated secondary data, and informed consent was waived.

## Conflicts of Interest

The authors declare no conflicts of interest.

## Supporting information


**Figure S1:** Week‐of‐year climatology of laboratory‐confirmed influenza detections and OTC respiratory medication sales. Values were standardized to facilitate comparison of seasonal patterns.

**Figure S2:** Mean cross‐correlation functions based on first‐differenced influenza detections and OTC sales. Negative lags indicate OTC sales changes preceding influenza changes.

**Figure S3:** Pairwise comparison of discrimination performance between forecasting models and the naïve persistence model using DeLong tests. Points represent differences in area under the receiver operating characteristic curve (AUC) relative to the naïve persistence model, with horizontal bars indicating 95% confidence intervals. Positive values indicate higher AUC than the naïve model, whereas negative values indicate lower AUC. Only statistically significant comparisons after Benjamini–Hochberg adjustment (*p* < 0.05) are shown.

**Figure S4:** Pairwise comparison of forecast accuracy between forecasting models and the naïve persistence model using Diebold–Mariano tests. Points represent the mean squared‐loss difference relative to the naïve persistence model, with horizontal bars indicating 95% confidence intervals. Negative values indicate lower forecast error (better performance) than the naïve model, whereas positive values indicate higher forecast error (poorer performance). Only statistically significant comparisons after Benjamini–Hochberg adjustment (*p* < 0.05) are shown.

**Table S1:** Composition of the OTC respiratory medication basket used for surveillance analyses.
**Table S2:** First‐difference sensitivity analysis of the lead–lag relationships between OTC respiratory medication sales and laboratory‐confirmed influenza detections by province.

## Data Availability

The data that support the findings of this study are not publicly available. Influenza surveillance data were obtained from the National Institute for Communicable Diseases (NICD), South Africa, and are subject to data sharing restrictions. Over‐the‐counter medication sales data were obtained from a commercial retailer and are subject to commercial confidentiality agreements. Aggregated data may be available from the corresponding author upon reasonable request and with permission from the respective data providers.
