# Supplementary material for: Assessing the Utility of Over‐The‐Counter Medication to Track Influenza Season Timing in a South African Population
Source: Influenza Other Respir Viruses. 2026 Sep 8;20(9):e70313. doi: 10.1111/irv.70313 (PMC13551350; doi:10.1111/irv.70313)
Supplement: Supplementary file 1 [file IRV-20-e70313-s001.pdf]

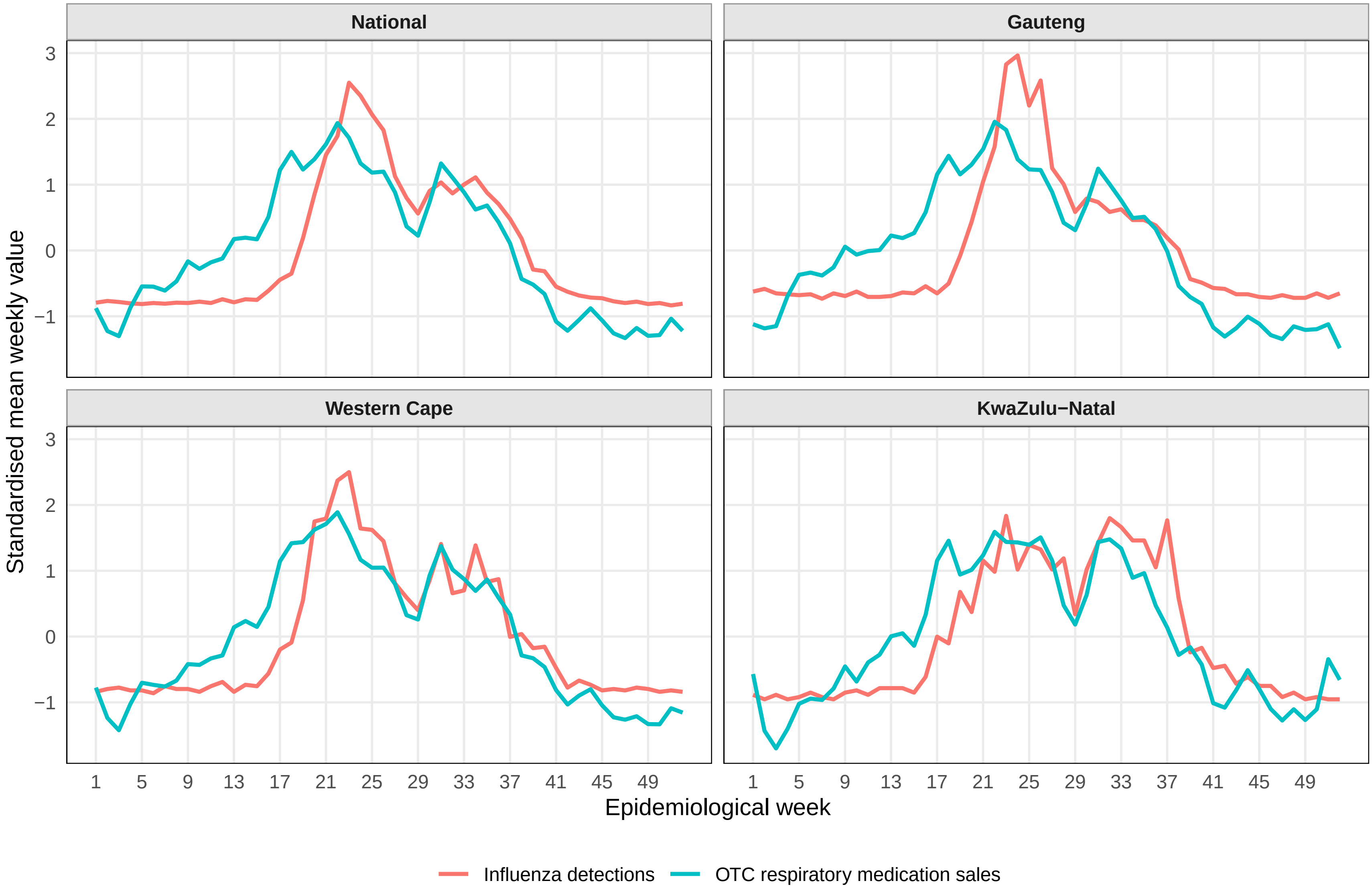
National
Gauteng
Western Cape
KwaZulu−Natal
Standardised mean weekly value
3
2
1
0
−1
1
5
9
13
17
21
25
29
33
37
41
45
49
Epidemiological week
Influenza detections
OTC respiratory medication sales
