# Supplementary material for: Assessing the Utility of Over‐The‐Counter Medication to Track Influenza Season Timing in a South African Population
Source: Influenza Other Respir Viruses. 2026 Sep 8;20(9):e70313. doi: 10.1111/irv.70313 (PMC13551350; doi:10.1111/irv.70313)
Supplement: Supplementary file 2 [file IRV-20-e70313-s002.pdf]

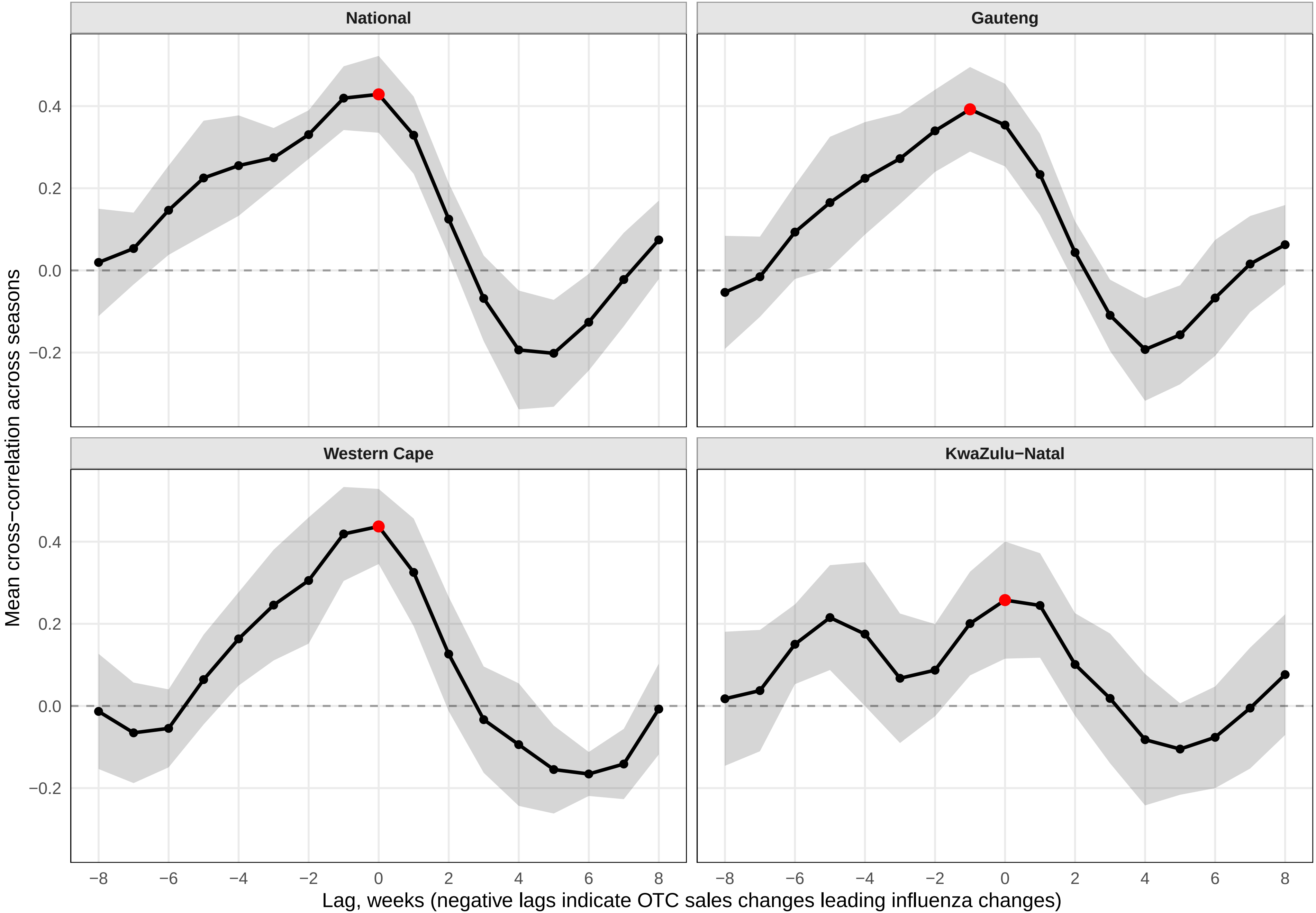
National
Gauteng
Western Cape
KwaZulu−Natal
Mean cross−correlation across seasons
0.4
0.2
0.0
−0.2
−8
−6
−4
−2
0
2
4
6
8
Lag, weeks (negative lags indicate OTC sales changes leading influenza changes)
