# Supplementary material for: Assessing the Utility of Over‐The‐Counter Medication to Track Influenza Season Timing in a South African Population
Source: Influenza Other Respir Viruses. 2026 Sep 8;20(9):e70313. doi: 10.1111/irv.70313 (PMC13551350; doi:10.1111/irv.70313)
Supplement: Supplementary file 3 [file IRV-20-e70313-s003.pdf]

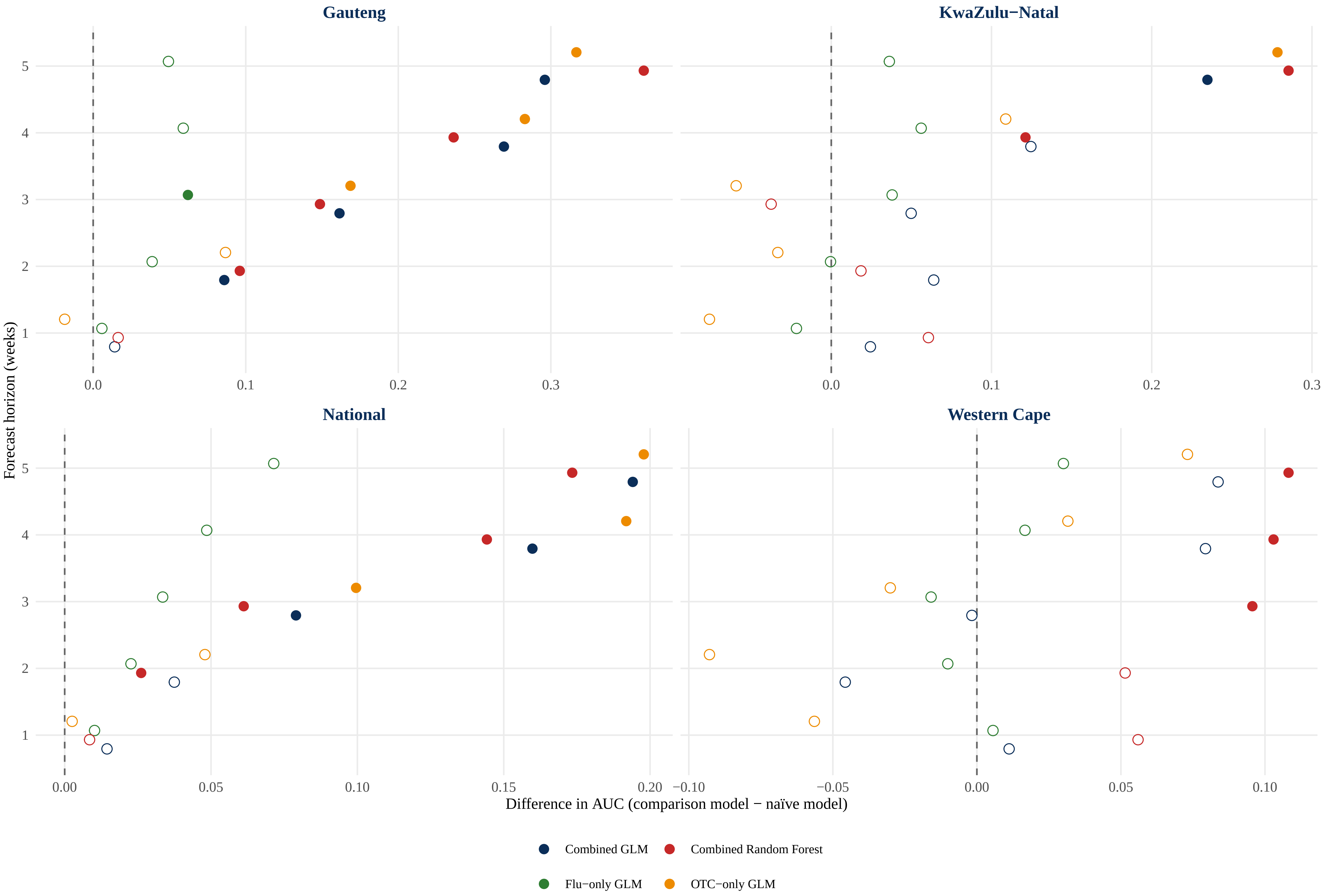

Filled points indicate BH−adjusted p < 0.05; open points indicate BH−adjusted p >= 0.05.
