# Supplementary material for: Assessing the Utility of Over‐The‐Counter Medication to Track Influenza Season Timing in a South African Population
Source: Influenza Other Respir Viruses. 2026 Sep 8;20(9):e70313. doi: 10.1111/irv.70313 (PMC13551350; doi:10.1111/irv.70313)
Supplement: Supplementary file 5 [file IRV-20-e70313-s005.docx]

Table S1. Composition of the OTC respiratory medication basket used for surveillance analyses

| **Therapeutic category** | **Description** | **Common active ingredients** | **Number of products** |
| --- | --- | --- | --- |
| Cold and influenza combination remedies | Multi-symptom products used for symptomatic relief of influenza and common cold symptoms, including fever, headache, nasal congestion and cough | Paracetamol, pseudoephedrine, phenylephrine, chlorpheniramine, antihistamines, vitamin C | 79 |
| Mucolytics and expectorants | Products used to reduce mucus viscosity and facilitate clearance of respiratory secretions | Acetylcysteine, bromhexine, guaifenesin | 65 |
| Cough suppressants (antitussives) | Products used for the management of dry or non-productive cough | Dextromethorphan, codeine phosphate, pholcodine | 52 |
| Productive cough and chest congestion remedies | Products intended for productive cough and chest congestion associated with respiratory infections | Bromhexine, guaifenesin, acetylcysteine, combination cough formulations | 57 |
| Nasal decongestants and sinus remedies | Products used to relieve nasal congestion and sinus symptoms | Pseudoephedrine, phenylephrine | 43 |
| Sore throat preparations | Lozenges and throat preparations used for symptomatic relief of sore throat | Menthol, antiseptics, local anaesthetics | 2 |
| Paediatric respiratory formulations | Respiratory symptom-relief products formulated specifically for children | Paediatric cough suppressants, expectorants, decongestants and cold-and-flu combinations | 7 |
| Bronchial and airway symptom preparations | Products marketed for bronchial irritation, airway mucus clearance and chest symptoms | Bromhexine, acetylcysteine and combination bronchial formulations | 14 |
| The OTC respiratory medication basket comprised 319 products. Product counts are approximate because several products contained multiple active ingredients and could be classified into more than one therapeutic category. The product basket remained unchanged throughout the study period. | | | |

Table S2. First-difference sensitivity analysis of the lead–lag relationships between OTC respiratory medication sales and laboratory-confirmed influenza detections by province.

| **Province** | **Seasons analysed** | **OTC sales led, n/N** | **Synchronous, n/N** | **Influenza leads, n/N** | **Lead range (weeks)** | **Mean lead (weeks)** | **Mean peak CCF (95% CI)** | **Median peak CCF** |
| --- | --- | --- | --- | --- | --- | --- | --- | --- |
| National | 10 | 7/10 | 2/10 | 1/10 | -5 to -1 | -2.57 | 0.491 (0.437; 0.544) | 0.479 |
| Gauteng | 10 | 8/10 | 2/10 | 0/10 | -5 to -1 | -2.38 | 0.464 (0.391; 0.536) | 0.429 |
| Western Cape | 10 | 6/10 | 2/10 | 2/10 | -5 to -1 | -2.17 | 0.503 (0.421; 0.584) | 0.520 |
| KwaZulu-Natal | 10 | 5/10 | 2/10 | 3/10 | -5 to -2 | -4.20 | 0.424 (0.355; 0.492) | 0.412 |
